# A voltage-based Event-Timing-Dependent Plasticity rule accounts for LTP subthreshold and suprathreshold for dendritic spikes in CA1 pyramidal neurons

**DOI:** 10.1007/s10827-024-00868-0

**Published:** 2024-03-12

**Authors:** Matus Tomko, Lubica Benuskova, Peter Jedlicka

**Affiliations:** 1 Centre of Biosciences, Institute of Molecular Physiology and Genetics, Slovak Academy of Sciences, Dubravska cesta 9, Bratislava, 840 05 Slovakia; 2 Faculty of Medicine, Institute of Medical Physics and Biophysics, Comenius University Bratislava, Bratislava, Slovakia; 3 Faculty of Mathematics, Physics and Informatics, Centre for Cognitive Science, Department of Applied Informatics, Comenius University Bratislava, Bratislava, Slovakia; 4 Faculty of Medicine, ICAR3R-Interdisciplinary Centre for 3Rs in Animal Research, Justus Liebig University Giessen, Giessen, Germany; 5 Institute of Clinical Neuroanatomy, Neuroscience Center, Goethe University Frankfurt, Frankfurt/Main, Germany

**Keywords:** Long-term potentiation, Dendritic spike, Compartmental model, CA1 pyramidal cell

## Abstract

Long-term potentiation (LTP) is a synaptic mechanism involved in learning and memory. Experiments have shown that dendritic sodium spikes (Na-dSpikes) are required for LTP in the distal apical dendrites of CA1 pyramidal cells. On the other hand, LTP in perisomatic dendrites can be induced by synaptic input patterns that can be both subthreshold and suprathreshold for Na-dSpikes. It is unclear whether these results can be explained by one unifying plasticity mechanism. Here, we show in biophysically and morphologically realistic compartmental models of the CA1 pyramidal cell that these forms of LTP can be fully accounted for by a simple plasticity rule. We call it the voltage-based Event-Timing-Dependent Plasticity (ETDP) rule. The presynaptic event is the presynaptic spike or release of glutamate. The postsynaptic event is the local depolarization that exceeds a certain plasticity threshold. Our model reproduced the experimentally observed LTP in a variety of protocols, including local pharmacological inhibition of dendritic spikes by tetrodotoxin (TTX). In summary, we have provided a validation of the voltage-based ETDP, suggesting that this simple plasticity rule can be used to model even complex spatiotemporal patterns of long-term synaptic plasticity in neuronal dendrites.

## Introduction

**Fig. 1:**
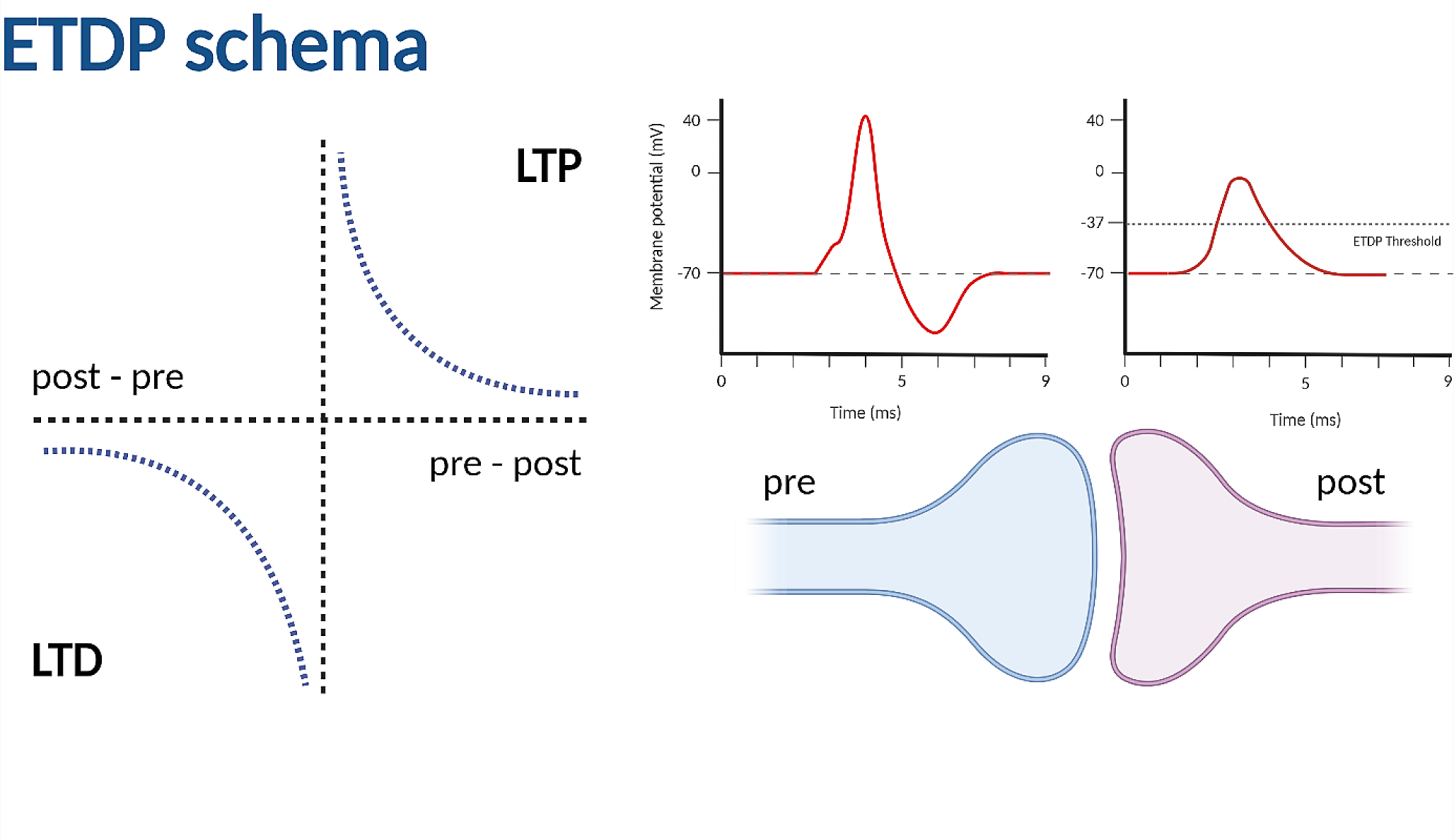
The Event-Timing-Dependent Plasticity rule. On the left is the nearest-neighbor implementation of ETDP, where each presynaptic event (pre) is paired with a postsynaptic event (post) that occurred before and one that occurred after the presynaptic event. The resulting synaptic weight change is the sum of the two. On the right, a presynaptic event is detected when a presynaptic spike or glutamate release occurs, and a postsynaptic event is detected when the EPSP at the site of the synapse exceeds a threshold value of -37 mV

LTP can be induced by a variety of stimulation protocols in a variety of conditions (Citri & Malenka, 2008). For instance, in the hippocampal CA1 neurons, theta-burst stimulation (TBS) applied to the entorhinal perforant pathway synapses at the distal apical tuft dendrites induces LTP that is strictly dependent on Na-dSpikes (Kim et al., 2015). Hence, we will refer to this form of LTP as a suprathreshold LTP with respect to the threshold for Na-dSpike initiation. Furthermore, subthreshold LTP has also been observed at synapses formed by the CA3 Schaffer collaterals on CA1 pyramidal cells under a low-frequency stimulation protocol. This protocol does not lead to the appearance of Na-dSpikes for a certain number of stimulated synapses, but LTP is still induced (Magó et al., 2020). In this short communication, we would like to show the potential of a single parsimonious principle, in which a postsynaptic plasticity threshold is crossed either by dSpikes or by spatio-temporal summation of cooperating synapses, to model these seemingly contradictory findings (Fig. 1).

## Methods

### Compartmental models of a CA1 pyramidal neuron

**Fig. 2:**
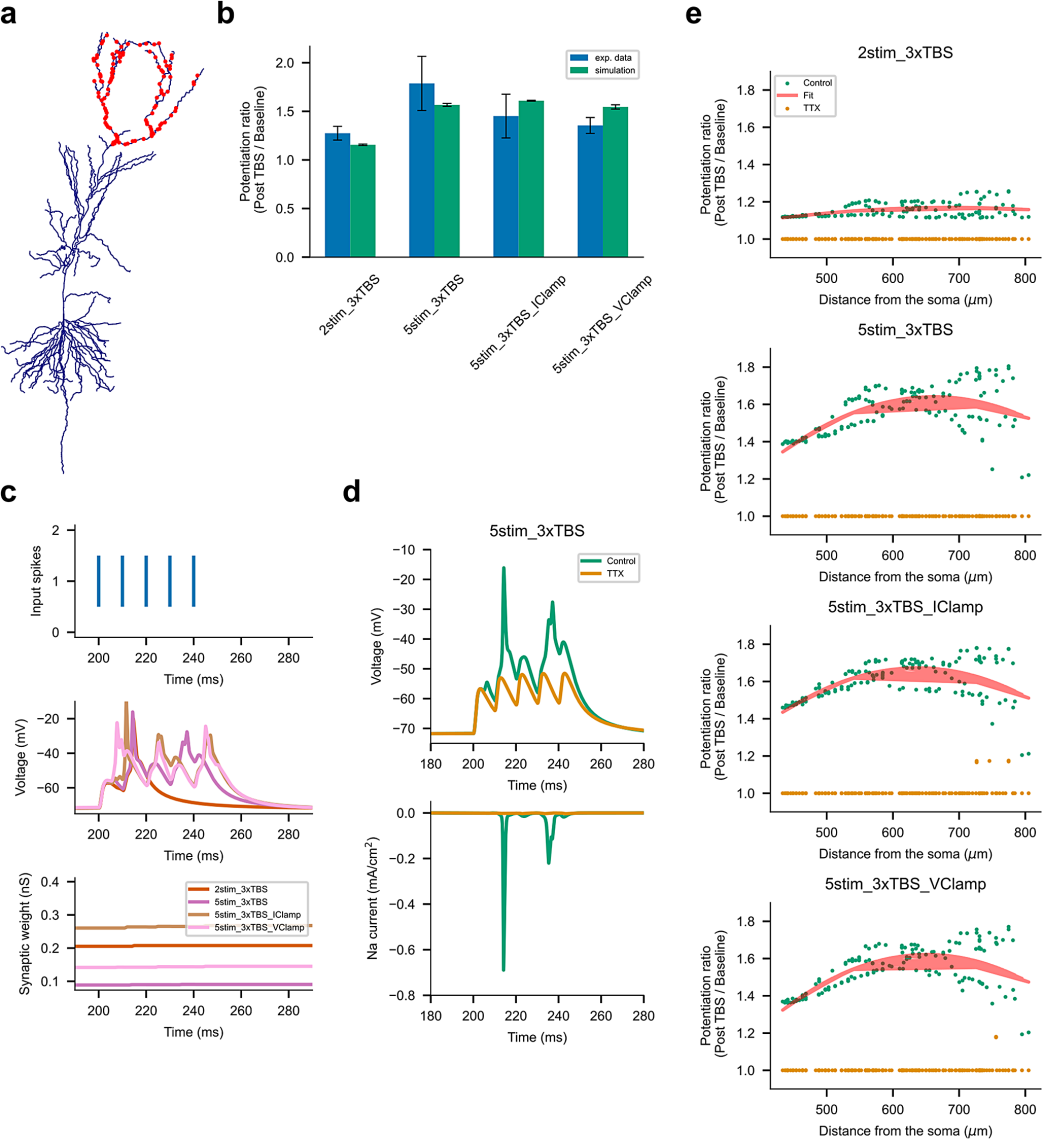
Simulations of suprathreshold LTP in apical tuft dendrites of the CA1 pyramidal cell. (**a**) Random placement of 150 excitatory synapses with AMPA and NMDA receptors along the apical tuft dendrites. (**b**) Comparison of the plasticity results obtained from the simulations of four different theta-burst stimulation (TBS) protocols with experimental results from Kim et al. (2015). The average LTP 1 min after TBS is shown as the mean of all synaptic weights ± SEM for each protocol. (**c**) Detailed analysis of the first burst under different stimulation protocols. Synaptic weight changes are shown as a function of time. (**d**) Effect of TTX on Na-dSpike generation. Sodium currents were measured from the same dendritic location in both control and TTX conditions. (**e**) Model predictions for LTP as a function of distance from the soma for different stimulation protocols, including those under TTX conditions

All simulations were performed using NEURON, which is embedded in Python 2.10 (Hines et al., 2009). The backward Euler method was used for numerical integration with a time step of 0.025 ms. Two full-morphology compartmental models of rat CA1 pyramidal cells (Kim et al., 2015; Magó et al., 2020) were used for the simulations of the LTP experiments. The simulation files of the Kim et al. (2015) CA1 model were downloaded from ModelDB (https://modeldb.science/), accession number 184054. We added 150 excitatory synapses, randomly distributed on the apical tuft dendrites of the model (Fig. 2a). While spines were not explicitly modelled, the associated surface area was accounted for by adjusting the specific membrane resistivity (R_m_) and specific membrane capacitance (C_m_) of compartments located more than 100 μm from the soma, which were multiplied by a factor of two. Each synapse was composed of an AMPA and an NMDA conductance, simulated by the sum of two exponential functions with rise time and decay time constants of 0.2 and 2 ms for AMPA (Katz et al., 2009) and 1 and 50 ms for NMDA (Spruston et al., 1995). Initial peak conductances were randomly selected from a lognormal distribution (mean 0.18, sigma 0.35 nS) for both AMPA and NMDA synapses (Rößler et al., 2023). The voltage-dependent magnesium block of the NMDAR was simulated using the equation: ${g}_{Mg}={\left[1+0.2801\times {Mg}_{ext}^{2+}\times exp\left(-0.062\times \left(V-10\right)\right)\right]}^{-1}$, where ${Mg}_{ext}^{2+}=1$ mM is the Mg^2+^ concentration in the bath and $V$ is the local dendritic voltage (Kim et al., 2015). In addition, we decided to modify the model by turning off the slow inactivation of Na_v_ channels, which resulted in a better fit to the experimental data. This modification is supported by a review of the literature and published models of CA1 cells (Bloss et al., 2018; Jarsky et al., 2005), as the presence or absence of slow inactivation in sodium channels varies across different neuronal models and experimental observations.

**Fig. 3:**
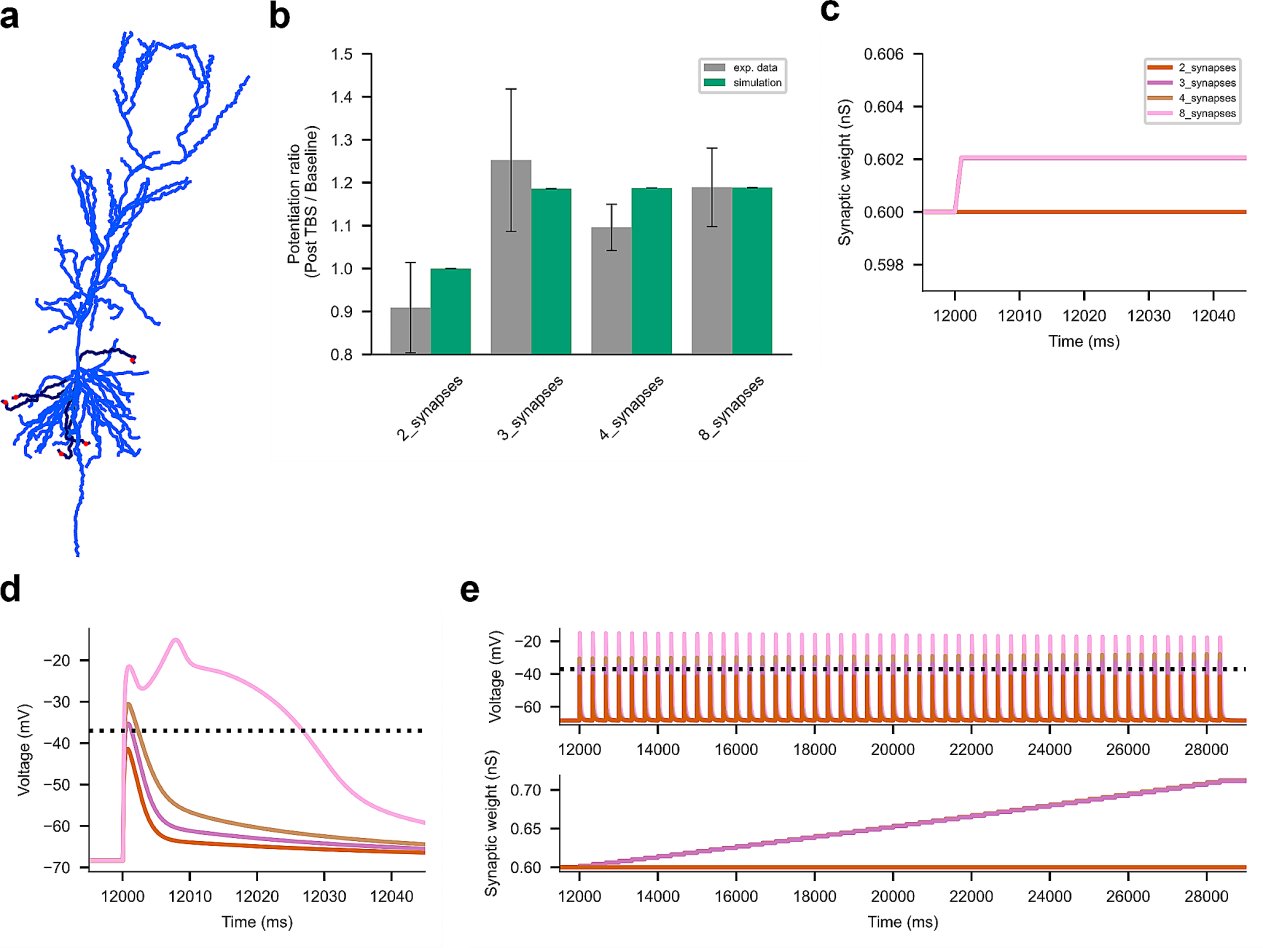
Simulations of the subthreshold and suprathreshold LTP in the perisomatic dendrites of the CA1 pyramidal cell. (**a**) Location of high-impedance dendritic spines with activated synapses (red dots) at the distal ends of highlighted perisomatic dendrites (dark blue). (**b**) Summary plot of induced LTP compared with experimental data from Magó et al. (2020) (mean ± SEM). (**c**) Evolution of synaptic weights of individual synapses for different scenarios. (**d**) Voltage traces from one spine head. (**e**) Dendritic voltage (top panel) with the − 37 mV threshold horizontal line and the evolution of the corresponding synaptic weights during the entire stimulation protocol

The model of Magó et al. (2020) from the ModelDB (accession number 265511) included synaptic conductances: AMPA had a rise time of 0.1 ms, a decay time of 1 ms, and a maximum conductance of 0.6 nS, while NMDA had a rise time of 2 ms, a decay time of 50 ms, and a maximum conductance of 0.8 nS. The voltage-dependent magnesium block was simulated using the equation ${g}_{Mg}={g}_{0}{\left(1+{Mg}_{ext}^{2+}/4.3\times exp\left(-0.071\times V\right)\right)}^{-1}$ where ${Mg}_{ext}^{2+}=1$ mM is the Mg^2+^ concentration in the bath and $V$ is the local dendritic voltage (Magó et al., 2020). In this model, synapses were placed on high-impedance dendritic spines consisting of a spine neck (length: 1.58 μm; diameter: 0.077 μm) and a spine head (length: 0.5 μm; diameter: 0.5 μm) with a total neck resistance of ~ 500MΩ (Harnett et al., 2012). Two, three, four or eight spines were placed on distal dendritic segments of 5 selected perisomatic dendrites (*x* = 0.96, Fig. 3a). To account for spines, C_m_ was increased, and R_m_ was decreased by a factor of 2 in dendritic compartments beyond 100 μm from the soma (Magó et al., 2020).

### ETDP synaptic plasticity rule

To model synaptic plasticity, the AMPA conductance representing synaptic weight was modified according to the ETDP synaptic plasticity rule (Fig. 1). In the ETDP, a presynaptic event is a presynaptic spike, while a postsynaptic event is detected when the voltage at the synaptic site exceeds the threshold of -37 mV (Jedlicka et al., 2015). Nearest-neighbor pairing was used to match presynaptic and postsynaptic events, where each presynaptic event was paired with a postsynaptic event that occurred before it and one after it. Weight change is calculated using the formula: $$w\left(t+\delta t\right)=w\left(t\right)\times \left(1+{\Delta }{w}_{p}-{\Delta }{w}_{d}\right)$$, where $${\Delta }{w}_{p}$$ is positive weight change (potentiation, LTP) and $${\Delta }{w}_{d}$$ is negative weight change (depression, LTD). Potentiation occurs when the presynaptic event preceded the postsynaptic event. Conversely, depression occurs when the postsynaptic event precedes the presynaptic spike. Thus, $${\Delta }{w}_{p}$$ and $${\Delta }{w}_{d}$$ are calculated according to the formula: $${\Delta }{w}_{p}\left({\Delta }t\right)={A}_{p}\times exp\left(-{\Delta }t/{\tau }_{p}\right)$$ if $${\Delta }t>0$$ and $${\Delta }{w}_{d}\left({\Delta }t\right)={A}_{d}\times exp\left({\Delta }t/{\tau }_{d}\right)$$ if $${\Delta }t<0$$, where $${\Delta }t={t}_{post}-{t}_{pre}$$, $${A}_{p}$$ and $${A}_{d}$$ are potentiation and depression amplitudes, respectively, $${\tau }_{p}$$ and $${\tau }_{d}$$ are decay constants for the time windows of plasticity. The values of $${A}_{p}=0.009$$$${A}_{d}=0.0012$$ for the TBS protocol, $${A}_{p}=0.0035$$, $${A}_{d}=0.001$$ for the LFS protocol and $${\tau }_{p}={\tau }_{d}=15 ms$$ for both protocols were optimized by hand. The model is very sensitive to the value of the postsynaptic event threshold, while LTP and LTD amplitudes and decay constants only influence the quantitative match.

### Stimulation protocols

For suprathreshold LTP, the 2stim_3xTBS stimulation protocol consisted of 3 trains of 2 pulses delivered at 100 Hz with a theta (5 Hz) interburst frequency repeated 3 times at 4 s intervals. The 5stim_3xTBS stimulation protocol consisted of 3 trains of 5 pulses delivered at 100 Hz with a theta (5 Hz) interburst frequency, repeated 3 times at 4 s intervals. Each 5stim_3xTBS protocol was simulated: (1) paired with brief (2 ms) somatic current injections at 50 Hz to elicit 3 action potentials during each burst (5stim_3xTBS_IClamp), (2) with the soma voltage clamped at − 70 mV (5stim_3xTBS_VClamp), or (3) alone (5stim_3xTBS) (Kim et al., 2015). The protocol used to induce subthreshold and suprathreshold LTP in perisomatic dendrites consisted of delivering 50 quasi-synchronous stimulations of selected spines (with a stimulus interval of 0.1 ms between spines) at a frequency of 3 Hz. Before and after the stimulation protocol, a set of four spines was stimulated separately, with a 200 ms interval between spines, and the trials were repeated at 0.5 Hz (Magó et al., 2020). Each simulation involved clustered spines on a single distal dendritic segment, with multiple simulations conducted for different dendrites to include the impact of spine positioning on synaptic plasticity.

## Results

### Simulations of suprathreshold LTP in apical tuft dendrites

Figure 2b summarizes the plasticity results from the compartmental model and compares them with the experimental results obtained by Kim et al. (2015), using the parameter configuration described above. The results of the simulations are given as averages of 5 runs (± SEM). The experimental results are taken from Fig. 6b of Kim et al. (2015) as average LTP from all synapses 1 min after TBS ± SEM. For all protocols, there was no significant difference *p* > 0.05 in the magnitude of LTP between simulated and experimental data. Student *t*-tests were performed using the NumPy and SciPy packages.

Figure 2c shows a detailed illustration of the first burst of TBS consisting of five presynaptic spikes delivered at a frequency of 100 Hz (top panel). For the 2stim_3xTBS protocol, only one Na-dSpike per one burst was detected as a postsynaptic event by the ETDP rule. The 5stim_3xTBS protocol elicited two Na-dSpikes while the 5stim_3xTBS protocol under somatic voltage clamp, i.e., 5stim_3xTBS_VClamp, elicited three Na-dSpikes per one burst. The 5stim_3xTBS_IClamp protocol involved three brief somatic current injections and also resulted in three Na-dSpikes per one burst. As in the experiment, there was no statistically significant difference between the average LTP induced by all these 5stim_3xTBS protocols (*p* > 0.05, one-way ANOVA). However, the 2stim_3xTBS protocol resulted in a significantly smaller LTP magnitude compared to the three 5stim_3xTBS (*p* < 0.05, one-way ANOVA).

In Fig. 2d, the top panel shows the local dendritic voltage for the 5stim_3xTBS, while the bottom panel shows the sodium current from the same dendritic location in both control and TTX conditions. In the control condition, two Na-dSpikes were observed, as evidenced by the inward sodium current. To simulate the effect of locally applied TTX, we reduced the sodium conductance in the dendrites by half. This manipulation prevented the induction of the Na-dSpike, as evidenced by the absence of significant changes in the dendritic voltage and sodium current under the TTX condition in agreement with Kim et al. (2015).

The predictions of the model are presented in Fig. 2e, which shows the distribution of potentiated synapses with the distance from soma. On average, we can see the strongest LTP in the middle part of the apical tuft based on a parabolic fit.

### Simulations of subthreshold and suprathreshold LTP in perisomatic dendrites

Using the low-frequency stimulation (LFS) protocol described in Methods, we modelled four scenarios in which we activated two, three, four, or eight synapses located at the distal ends of perisomatic dendrites (red dots on highlighted branches in Fig. 3a). The resulting LTP shown in Fig. 3b (green bars) represents the average LTP across all simulations for a given scenario and comparison with the experimental data of Magó et al. (2020) (the seventh minute after HFS ± SEM from Fig. 3e for 3 or 4 synapses and from Fig. 5b for 8 synapses, grey bars). The results showed that LTP was induced when three or more synapses were activated, which is consistent with the results of Magó et al. (2020). For all numbers of spines, there was no significant difference in the magnitude of LTP between simulated and experimental data (Student’s t-test, *p* > 0.05). Figure 3c and d show that when three or more synapses were stimulated, the voltage at the spine head exceeded the postsynaptic event threshold and triggered ETDP, leading to an increase in synaptic weights even in the absence of a Na-dSpike. Only the activation of a cluster of eight synapses was sufficient to induce the dSpike. Figure 3e shows the dendritic voltage and the evolution of the synaptic weights throughout the stimulation protocol demonstrating an increase in voltage amplitude with increasing synaptic weights. Taken together, the simulation results validate the simple ETDP model for subthreshold and suprathreshold LTP induction in perisomatic dendrites.

## Discussion

To account for the experimental data showing subthreshold and suprathreshold LTP for Na-dSpike, we proposed a simple synaptic plasticity rule based on the timing of local pre- and postsynaptic events. First, we applied it to the experimental data of Kim et al. (2015), which demonstrated the requirement of Na-dSpikes for the occurrence of LTP at the distal apical tuft dendrites of CA1 pyramidal cells. By implementing the same stimulation protocols, we successfully replicated the observed LTP, supporting the effectiveness of the ETDP model. Interestingly, our model showed that bAPs had minimal influence on LTP at distal parts of apical tuft synapses due to the strong voltage attenuation of bAPs, in agreement with the experimental findings.

We then simulated synaptic plasticity at synapses located on perisomatic oblique dendrites of CA1 pyramidal cells. In this particular region, both subthreshold and suprathreshold LTP has been observed with respect to Na-dSpikes (Magó et al., 2020). Using the ETDP model, we were able to capture the dynamics of synaptic plasticity as a result of functional clusters of activated synapses: no LTP with 2 synapses, subthreshold LTP with 3 or 4 synapses, and suprathreshold LTP with 8 synapses, mirroring the magnitudes observed in the data of Magó et al. (2020).

To our best knowledge, these data have not been modelled before. There are several other, biophysically and/or biochemically more sophisticated models of long-term synaptic plasticity involving local postsynaptic voltage that could reproduce the data (Clopath et al., 2010; Ebner et al., 2019; Mäki-Marttunen et al., 2020). However, according to the principle of parsimony our model is the simplest possible one. Indeed, the goal of our simulations was to find the biophysically minimalist plasticity model associated with a dendritic voltage threshold that could be responsible for explaining the observed synaptic changes. This does not mean other factors and processes are not important, it simply means they do not seem to play an explanatory role in the present context.

## Data Availability

Code and data of this work are available at https://github.com/tomko-neuron/ETDP-CA1 and https://zenodo.org/doi/10.5281/zenodo.8348966.
